# Prospective, Open-Label, Multi-Centre, Randomized Study to Compare the Effectiveness, Safety, and Tolerability of Lulican™ Shampoo Versus Ketoconazole Shampoo in Indian Adult Patients With Mild to Moderate Scalp Seborrheic Dermatitis (LEAD Study)

**DOI:** 10.7759/cureus.32035

**Published:** 2022-11-30

**Authors:** Srichand G Parasramani, Vishalakshi Vishwanath, Deepti Ghia, Miti R Gandhi, Dhiraj Dhoot, Hanmant Barkate

**Affiliations:** 1 Dermatology, Anisha Clinic, Mumbai, IND; 2 Dermatology, Rajiv Gandhi Medical College and Chhatrapati Shivaji Maharaj (CMS) Hospital, Mumbai, IND; 3 Dermatology, South Mumbai Dermatology Clinic, Mumbai, IND; 4 Global Medical Affairs, Glenmark Pharmaceuticals Limited, Mumbai, IND; 5 Global Medical Affairs, Glenmark Pharmaceutical Limited, Mumbai, IND; 6 Global Medial Affairs, Glenmark Pharmaceuticals Limited, Mumbai, IND

**Keywords:** effectiveness, scalpdex 23, quality of life, sd, ketoconazole, seborrhic dermatitis, luliconazole

## Abstract

Introduction

Although seborrheic dermatitis (SD) is not lethal, it has a significant impact on the quality of life. Many cases of SD are managed with ketoconazole, but luliconazole has shown an equivalent or lower minimum inhibitory concentration (MIC), but not many studies have been done for its efficacy and safety in SD. With this in mind, we set out to conduct a study comparing the effectiveness, safety, and tolerability of Lulican™ (luliconazole 1% + salicylic acid 3% + ZPTO 1%) shampoo and Ketoconazole (Ketoconazole 2% + ZPTO 1%) shampoo in the treatment of SD.

Materials and methods

In this prospective, randomized, multi-center study, mild to moderate scalp SD patients were prescribed Lulican™ or Ketoconazole shampoo three times a week for a duration of four weeks. Effectiveness assessment was done with the Seborrheic-Dermatitis-Severity-Score (SDSS) and Physician-Global-Assessment (PGA), and quality of life was assessed with the help of the Scalpdex-23 questionnaire.

Results

At four weeks, 68% and 57.9% reduction was seen in SDSS in Lulican™ and Ketoconazole shampoo, respectively. Moreover, 58% and 44% of patients achieved excellent to moderate responses as per PGA with Lulican™ and ketoconazole shampoo, respectively. For safety, no statistical difference was reported, but product tolerability and subjective cosmetic acceptability were significantly better in the Lulican™ group as compared to the Ketoconazole group at the end of four weeks. The mean Scalpdex-23 score at week four was reduced by 35.7% and 21.1% in Lulican™ and ketoconazole groups, respectively (p<0.05).

Conclusion

While both treatments were successful in alleviating SD symptoms and were well tolerated, Lulican™ stood out as a preferred treatment option due to better quality of life (QoL) improvement in SD.

## Introduction

Pityriasis sicca, or dry, flaky scales, is one form of seborrheic dermatitis (SD) of the scalp, whereas seborrhoea, or greasy, crusty scales, is another. Even though seborrheic dermatitis is not lethal, it has a significant impact on the quality of life of those who suffer from it [1]. A poll estimates that almost 50 million Americans have seborrheic dermatitis and that nearly $300 million is spent each year on dandruff treatments. According to a study conducted in India on children younger than five years old, the prevalence of seborrheic dermatitis peaked in infancy and gradually declined thereafter. In adults in India, 18.7% of instances of scalp dermatoses were shown to be attributable to SD. Approximately 50-90% of psoriasis cases and up to 95% of SD cases involve the scalp, according to various sources [2-4]. Therefore, the quality of life of many individuals is negatively impacted by scalp dermatitis. As a result, scalp care calls for long-term approaches after comparing the benefits of keeping the illness in remission and the risks of using many medicines over time [5].

Although the exact cause of SD has yet to be determined, it appears to be a complex disease with a link to decreased sebaceous gland activity and the presence of skin yeasts belonging to Malassezia spp. Malassezia yeasts [6,7] function by breaking down sebaceous triglycerides to release free fatty acids, which then aggravate inflammation in those who are already predisposed to it [8-9]. Most cases of SD can be treated with shampoos containing anti-yeast medicines such as ketoconazole or zinc pyrithione (ZPTO). Salicylic acid-containing treatments can be beneficial for severe scaling, while selenium sulphide or tar-containing shampoos have also been commonly utilised. However, its use for the treatment of SD is limited by several important factors, including the high incidence of resistance to the azole treatment, recurrent rates, toxicity associated with the constant use of anti-fungal agents (contact dermatitis in the case of ketoconazole), and the price of anti-fungal agents [10]. However, Uchida et al. observed in their investigation that the minimum inhibitory concentration (MIC) of luliconazole against *M. restricta* (considered a pathogenic factor in seborrheic dermatitis) was nearly equivalent to or lower than ketoconazole, the medicine most widely used in the management of Malassezia infections [11-13]. In addition, luliconazole is three to four times more powerful than bifonazole and terbinafine against *M. furfur*, *M. sympodialis*, and *M. slooffiae*, and its efficacy is about on par with that of lanoconazole.

Though many clinical studies have demonstrated the safety of luliconazole [14-16], its effectiveness or safety in the treatment of seborrheic dermatitis of the scalp has not been evaluated to date. With this in mind, we set out to conduct a study comparing the effectiveness, safety, and tolerability of Lulican™ (luliconazole 1% + salicylic acid 3% + ZPTO 1%) shampoo and ketoconazole (ketoconazole 2% + ZPTO 1%) shampoo in the treatment of patients with seborrheic dermatitis of the scalp.

## Materials and methods

We planned a prospective, randomized, multi-centre study to compare the effectiveness, safety, and tolerability of Lulican™ versus Ketoconazole+ZPTO, in patients suffering from mild to moderate seborrheic dermatitis of the scalp. Following the ethics committee's approval, the trial was prospectively registered (CTRI 2021/09/036865) and conducted in accordance with the Good Clinical Practice guidelines and the Declaration of Helsinki 1996. Patients attending the dermatology clinics at three different locations with chief complaints of mild to moderate seborrheic dermatitis of the scalp were screened for inclusion in the study. The recruitment period was extended from October 2021 to March 2022. Inclusion criteria consisted of: (i) age >18 years; (ii) a two-week washout from topical antifungals/corticosteroids; (iii) a four-week washout from oral antifungals/corticosteroids and hormonal therapy. Exclusion criteria on the other hand comprised of: (i) patients with a history of sensitivity or allergic reaction to any components of the drug and (ii) pregnant females or those planning pregnancy. Written informed consent was obtained from each of the study participants before enrolment in the study. A randomization list was generated with the help of a validated system that automated the random assignment of the study participants into two treatment groups in 1:1 ratio. Patients in group I were given Lulican™ shampoo (luliconazole 1%+ salicylic acid 3%+ ZPTO 1%), while patients randomised to group II were given combination ketoconazole shampoo (ketoconazole 2% and ZPTO 1%) of a generic brand. Both drugs were supplied by Glenmark Pharmaceuticals, Ltd. (Mumbai, India). For four weeks, all patients used shampoo three times per week.

Clinical evaluation

The primary endpoint was efficacy assessment at weeks 2 and 4 as compared to the baseline. The secondary endpoint was the evaluation of tolerability and cosmetic acceptability along with safety and QoL assessment. Efficacy assessment was done by computing the Seborrheic Dermatitis Severity Score (SDSS) at baseline, at two weeks, and at four weeks post-randomization (Table 1). The total score was calculated based on the individual scores of the symptoms, giving a minimum score of 0 and a maximum score of 16. Moreover, a Physician Global Assessment (PGA) was conducted at the end of the study using a six-point scale that measured as follows: (i) complete response (>90% improvement); (ii) excellent response (70-90% improvement); (iii) moderate response (40-69% improvement); (iv) mild response (<40% improvement); (v) no response (no change), and (vi) worsening. In addition to this, an investigator evaluation of Lulican™ and ketoconazole + ZPTO tolerability was carried out with the help of a four-point scale: 0 = very poor, 1 = poor, 2 = good, and 3 = excellent. Also, subjective cosmetic acceptability was evaluated at the end of week 4 on a 3-point scale: 0 = poor, 1 = good, and 2 = excellent. In addition, quality of life was assessed with the help of the Scalpdex 23 questionnaire, which consists of 23 questions based on three domains (symptoms, emotion, and functioning). All the questions were included, and the evaluation was based on three domains. Each domain had a score ranging from 0 to 100, wherein the results were interpreted as follows: 0: never; 25: rarely; 50: sometimes; 75: often; and 100: all the time.

**Table 1:** Symptom Scale of Seborrheic Dermatitis Severity Score

Score	Scalp area affected (%)	Erythema	Scaling	Itching
0	0	None	None (no desquamation)	None
1	<25%	Mild	Mild (few small loose white flakes)	Mild
2	26-50%	Moderate	Moderate (several small loose white flakes)	Moderate
3	51-75%	Severe	Severe (several small to large loose white flakes)	Severe
4	>75%	Very severe	Very severe (many large adherent white flakes)	Very severe

Statistical analysis

Results were presented as mean scores, and the therapeutic efficacy in both groups was evaluated. Both paired and unpaired t-tests were used for the clinical severity score. A paired t-test was used to compare the group at two and four weeks, whereas an unpaired t-test was used to compare the groups. The difference in the proportion of patients with a change in mean scores (based on improvement criteria) was analysed using the Chi-square test. Data were analysed using the IBM SPSS (Statistical Package for Social Sciences, IBM Corp., Armonk, NY) statistics version 20. A p-value of < 0.05 was considered to be statistically significant.

## Results

A total of 72 patients were recruited and completed the study over a period of six months, from October 2021 to March 2022. The mean age of participants in the Lulican™ shampoo group was 31.47±13.43, while that of the study participants in the ketoconazole + ZPTO shampoo group was 26.69±9.96. The baseline demographics, along with the Seborrheic Dermatitis Severity score and the Scalpdex 23 score, are represented in Table 2.

**Table 2:** Demographic characteristics of the study participants

	Lulican™shampoo	Ketoconazole + ZPTO shampoo	P-value
N	36	36	
Male	12	21	
Female	24	15	
Mean age in years	31.47±13.43	26.69±9.96	0.09
Duration of disease in months (Median)	11 (1–120)	12 (1–300)	0.41
Baseline Seborrheic Dermatitis Severity score (mean ± SD)	9.14±2.21	9.17±2.55	0.9
Baseline Scalpdex 23 score (mean ± SD)	52.42±13.91	51.58±16.67	0.8

Effectiveness assessment

At the end of two weeks, the mean SDSS in the Lulican™ group was 5.00 ± 1.66 while in the ketoconazole + ZPTO group, it was 5.69 ± 2.56, accounting for a 45.3% and a 37.9% reduction in the baseline seborrheic dermatitis severity score in both the groups, respectively. This change was statistically significant in both groups as compared to the baseline. At the end of four weeks, SDSS further reduced to 2.92 ± 2.56 (68% reduction from baseline) and to 3.86 ± 2.80 (57.9% reduction from baseline) in the Lulican™ and ketoconazole + ZPTO groups, respectively. These findings were statistically significant in both groups in comparison with the baseline. However, there was no statistically significant difference between the Lulican™ shampoo group and the ketoconazole + ZPTO shampoo group on the intergroup comparison, at the end of four weeks (Table 3).

**Table 3:** Effectiveness and safety assessment in both the groups at different time intervals

Assessment	Parameters	Week 2			Week 4		
		Lulican ™shampoo	Ketoconazole +ZPTO shampoo	p-value	Lulican™shampoo	Ketoconazole +ZPTO shampoo	p-value
Effectiveness assessment	Seborrheic Dermatitis Severity Score (mean ± SD)	5.00±1.66	5.69±2.56	0.17	2.92±2.56	3.86±2.80	0.14
Safety assessment	Product tolerability assessment	2.61±0.49	2.28±0.66	0.01	2.78±0.42	2.28±0.70	5 × 10^−^^4^
	Subjective cosmetic acceptability	2.69±0.47	2.19±0.71	8E-04	2.81±0.40	2.17±0.74	1 × 10^−^^4^
QoL assessment	Scalpdex 23 score (mean ± SD)	NA	NA		33.67±11.39	40.67±13.19	0.01

Furthermore, 21 patients (58%) in the Lulican™ shampoo group and 16 patients (44%) in the ketoconazole + ZPTO shampoo group achieved excellent to moderate response as per PGA (p=0.34). Fourteen patients (39%) in the Lulican™ group and 18 patients (50%) in the ketoconazole + ZPTO group reported having mild to moderate response (0.47) (Table 4).

**Table 4:** Number of patients achieving Physician-Global-Assessment response

PGA response	Lulican^TM ^ shampoo (N)	Ketoconazole + ZPTO shampoo (N)
Complete	8	6
Excellent	13	10
Moderate	12	15
Mild	2	3
No	1	2
Worsened	0	0

Safety assessment

In the Lulican™ group, a total of five patients reported adverse events while in the ketoconazole + ZPTO group, AE was reported by two patients only (p = 0.43) (Table 5). However, in terms of product tolerability and subjective cosmetic acceptability, there was a statistically significant difference between both groups at weeks 2 and 4. In the Lulican™ group, both product tolerability and subjective cosmetic acceptability showed improvement at week 4 from week 2, whereas in the ketoconazole + ZPTO group, there was no improvement in either of the assessments at week 4 in comparison to week 2. However, both product tolerability and subjective cosmetic acceptability were significantly better in the Lulican™ group as compared to the ketoconazole + ZPTO group at the end of week 4 (p < 0.0005 and p < 0.0001, respectively) (Table 3).

**Table 5:** Number of patients with adverse events in both the groups at the end of four weeks

	Lulican^TM ^shampoo (N)	Ketoconazole + ZPTO shampoo (N)
Dryness of scalp	2	2
Hair loss	2	0
Increased flaking	1	0

QoL assessment

The Mean Scalpdex 23 score reduced to 33.67 ± 11.39 at week 4 from a baseline score of 52.42 ± 13.91 in the Lulican™ group (35.7% reduction) whereas, in the ketoconazole + ZPTO group, it was reduced to 40.67 ± 13.19 from a baseline score of 51.58 ± 16.67 (21.1% reduction). This change in the mean Scalpdex score at the end of four weeks was statistically significant for both groups as compared to baseline; however, it was also statistically significant (p<0.005) for the Lulican™ group as compared to ketoconazole + ZPTO group at the end of four weeks (Table 3).

Moreover, the scalp-related quality of life was most commonly affected by subjective scalp itching since the symptom domain consisting of "my scalp itches" had a score of 63.19 (28.96) in the Lulican™ group and 60.42 (22.66) in the ketoconazole + ZPTO group. Quality of life was least affected by "my scalp bleeds," with a score of 18.75 (22.66) in the Lulican™ group and 13.57 (23.75) in the ketoconazole + ZPTO group. The mean scores for all items in different domains are listed in Table 6. Though all Scalpdex items improved in both groups, a statistical difference was noted in almost all items for the Lulican™ group over the ketoconazole + ZPTO group suggesting better QoL with Lulican™ group.

**Table 6:** Scalpdex items, in the order, presented to patients* in both groups *The scale scores of symptom (S), emotions (Em), and functioning (F) are calculated by the average of the item scores that pertain to that particular scale. The items are scored on a scale from 0 to 100 (0 indicates never; 25, rarely; 50, sometimes; 75, often; and 100, all the time).

Item		Hypothesized construct	Lulican^TM^; mean ± SD score		Ketoconazole + ZPTO; mean ± SD score	
			Baseline	Week 4	Baseline	Week 4
1	My scalp hurts	S	23.61±28.63	7.86±15.78	20.83±26.39	9.72±18.20
2	My scalp condition makes me feel depressed	Em	25.00±23.15	12.14±19.53	24.31±23.52	15.28±16.12
3	My scalp itches	S	63.19±28.96	24.29±27.44	60.42±22.66	29.86±26.61
4	I am ashamed of my scalp condition	Em	29.86±30.95	8.57±19.12	26.39±26.69	18.06±19.47
5	I am embarrassed by my scalp condition	Em	29.17±25.70	11.43±20.42	26.39±28.00	15.97±20.83
6	I am frustrated by my scalp condition	Em	38.19±28.34	20.00±22.52	38.19±31.90	21.53±24.02
7	I am humiliated by scalp condition	Em	23.61±28.00	6.43±16.43	20.83±27.71	14.58±21.02
8	My scalp condition bleeds	S	18.75±22.66	5.71±14.96	13.57±23.75	8.57±15.98
9	I am annoyed by scalp condition	Em	47.92±24.18	20.00±24.10	38.19±28.34	25.00±26.73
10	I am bothered by the appearance of scalp condition	Em	38.19±24.99	12.86±22.99	34.03±21.67	25.00±24.64
11	My scalp condition makes me feel self-conscious	Em	34.72±27.57	15.71±21.93	31.25±28.27	17.36±18.73
12	I am bothered that my scalp condition is incurable	Em	39.58±30.69	20.00±21.69	29.86±27.92	18.06±20.36
13	My scalp condition affects how I wear my hair (hairstyle, hats)	F	27.08±31.27	10.00±19.36	26.39±30.44	13.19±19.35
14	I am bothered by people's questions about my scalp condition	Em	19.44±25.43	7.86±14.57	23.61±26.01	15.97±21.67
15	My scalp condition affects the color of clothes I wear	F	20.83±30.76	2.14±9.34	20.83±31.34	13.89±21.91
16	I am bothered by the persistence/recurrence of my scalp condition	Em	48.61±26.69	22.86±27.37	47.92±22.66	31.94±25.08
17	I feel stressed about my scalp condition	Em	30.56±27.46	12.86±21.33	38.89±28.94	21.53±22.48
18	Caring for my scalp condition is inconvenient for me	F	31.94±28.42	11.43±21.30	29.86±27.27	18.06±20.36
19	I feel that my knowledge about caring for my scalp is adequate	Em	34.72±26.91	23.57±31.47	38.19±30.17	30.56±30.54
20	The cost of caring for my scalp condition bothers me	Em	33.33±30.47	14.29±22.10	31.25±31.83	17.36±21.40
21	My scalp condition makes my daily life difficult	F	26.39±26.01	11.43±21.30	31.94±29.04	22.92±28.27
22	My scalp condition makes me feel different from others	Em	22.22±25.90	10.14±18.53	22.22±25.20	17.36±23.01
23	My scalp condition makes it hard to go to the hairdresser	F	27.08±30.10	7.14±17.75	25.00±32.18	15.00±22.85

## Discussion

Over the years, many approaches for treating seborrheic dermatitis of the scalp have been explored. After ketoconazole was proven to be a successful treatment for SD, most studies focused on comparing other antifungal medications to ketoconazole. The ideal anti-fungal agent would be one that effectively suppressed illness over a long period of time while having limited potential for causing unwanted effects with continuous use [17]. Several antifungal drugs, such as zinc pyrithione, ketoconazole, bifonazole, miconazole, terbinafine, and ciclopirox olamine, have been studied as possible therapies for seborrheic dermatitis. However, it has been proven that weekly preventive usage of ketoconazole shampoo can clear up seborrheic dermatitis on the scalp [18]. For their basic cleansing qualities, which remove lipids (the substrate for Malassezia spp.), the surfactants included in shampoo bases are well-known to be effective in the treatment of dandruff and other scalp and hair conditions. Patients also benefit from the mechanical massage of the scalp that occurs during hair washing, which aids in the breakdown and removal of scales [19]. In fact, moderate cases of the condition can be managed by regular shampooing with non-medicated shampoos.

However, topical ketoconazole has its own disadvantages, including contact dermatitis, rashes, and medication interactions with the CYP3A4 enzymes if used over an extended period [20-21]. Kubicki et al. reported a case of non-scarring hair loss secondary to androgenic alopecia who developed pink discolouration of the hair with 2% ketoconazole shampoo [22]. Another study by Ahmed et al. found that ketoconazole shampoo was associated with contact dermatitis in 10% of the study participants [23]. However, luliconazole’s lack of serious adverse effects is encouraging. Jones et al. found that 1% luliconazole cream had a lower incidence of adverse events than vehicles (16.9 percent) [14]. Even with a 1% concentration, luliconazole cream was found to have few adverse effects, as described by Watanabe et al. [15]. Due to this, we aimed to conduct this study to compare luliconazole and ketoconazole shampoos in SD.

Luliconazole is an azole that is highly effective against fungal infections. Strong in vitro antifungal activity and favourable pharmacokinetic qualities in the skin may account for luliconazole’s powerful antifungal efficacy [24]. Study participants assigned to the Lulican™ group showed significant improvement in all four domains assessed over a four-week period compared to those assigned to the ketoconazole group: (i) seborrheic dermatitis severity score; (ii) product tolerability assessment; (iii) subjective cosmetic acceptability; and (iv) Scalpdex 23 score for quality of life. This is because luliconazole has a higher safety and tolerability profile as compared to ketoconazole, and because patients who were given the Lulican™ shampoo found it more acceptable. A clinical trial for the evaluation of the quality of life associated with the use of ketoconazole demonstrated that 35% of the patients were dissatisfied with ketoconazole treatment at the end of four weeks [25]. These findings were like those seen in our study.

Seborrheic dermatitis can significantly reduce a patient's quality of life, which can result in diminished self-worth, a poor social reputation, and a lack of confidence, primarily as a result of scalp scales. Because seborrheic dermatitis causes flakes to shed, which may be mistaken for filth and eventually induce social isolation, it can be uncomfortable. The person's identity may be altered as a result, which could lower self-esteem and have a detrimental effect on the quality of life. In a study conducted in Thailand by Araya Manapajon, 166 seborrheic dermatitis patients' clinical features and quality of life were assessed. They came to the conclusion that patients with seborrheic dermatitis had higher mean dermatology quality life index (DQLI) scores, particularly those who were young, female, and had significant scalp lesions. [26] In a similar way, Szepietowski et al. conducted a study on 3000 Polish patients with seborrheic dermatitis and found that the condition had a considerable and detrimental impact on the patients' quality of life [27]. Even in this study, females and younger patients with seborrheic dermatitis had a worse quality of life than the other patients as a result of the condition. Oztaz et al. have even raised the possibility that seborrheic dermatitis may increase the risk of developing depression [28]. According to a study by Peyri et al., individuals with mild or moderate seborrheic dermatitis had a considerably higher quality of life than those with a severe or very severe condition, proving that the severity of the disease affects QoL [29]. Scaling scalps, erythematous patches on the lips, ears, sternum, nasolabial creases, eyebrows, and eyelids are all symptoms of SD. SD can coexist with emotional symptoms such as depression, anxiety, and others and has negative consequences for patients' quality of life (QoL) [30].

The quality of life in the case of study participants randomised to the Lulican™ group was significantly better than that of those who received ketoconazole shampoo. The mean Scalpdex 23 score at four weeks was 33.67 ± 11.39 in the Lulican™ group, whereas it was 52.42 ± 13.91 in the ketoconazole group. Lulican™ shampoo, by improving the patient's quality of life, not only reduces the disease severity and intensity of the lesions but also renders the patients of SD confident enough to interact freely with their peers, relatives, and in their social circle. In addition to its role in SD, luliconazole may also reduce the incidence of depression, anxiety, and other emotional disturbances. Quality of life with Lulican™ shampoo not only has a better clinical outcome but also improves the overall patient acceptability of the drug, thereby improving the clinical outcomes of SD. The only limitation of our study was the small sample size and the smaller number of study sites. However, this can be addressed by conducting a clinical trial on a larger scale that includes a larger sample size of patients suffering from SD.

## Conclusions

Although in vitro studies have shown potential for luliconazole in the therapy of SD, there have been no human clinical trials of this medication so far. To the best of our knowledge, this is the first study to compare the efficacy, tolerability, and quality of life of luliconazole and ketoconazole shampoos for the treatment of SD. While both were successful in alleviating SD symptoms, Lulican™ stands out as a preferred treatment option due to better QoL improvement in SD.
